# Nonlinear dynamics of trions under strong optical excitation in monolayer MoSe_2_

**DOI:** 10.1038/s41598-018-20810-6

**Published:** 2018-02-05

**Authors:** Jialiang Ye, Tengfei Yan, Binghui Niu, Ying Li, Xinhui Zhang

**Affiliations:** 1 State Key Laboratory of Superlattices and Microstructures, Institute of Semiconductors, Chinese Academy of Sciences, Beijing, 100083 P. R. China; 2 College of Materials Science and Opto-Electronic Technology, University of Chinese Academy of Sciences, Beijing, 100049 P. R. China

## Abstract

By employing ultrafast transient reflection measurements based on two-color pump-probe spectroscopy, the population and valley polarization dynamics of trions in monolayer MoSe_2_ were investigated at relatively high excitation densities under near-resonant excitation. Both the nonlinear dynamic photobleaching of the trion resonance and the redshift of the exciton resonance were found to be responsible for the excitation-energy- and density-dependent transient reflection change as a result of many-body interactions. Furthermore, from the polarization-resolved measurements, it was revealed that the initial fast population and polarization decay process upon strong photoexcitation observed for trions was determined by trion formation, transient phase-space filling and the short valley lifetime of excitons. The results provide a basic understanding of the nonlinear dynamics of population and valley depolarization of trions, as well as exciton-trion correlation in atomically thin MoSe_2_ and other transition metal dichalcogenide materials.

## Introduction

Monolayers of transition metal dichalcogenides (TMDCs), such as MoS_2_ and WSe_2,_ are direct-band-gap semiconductors with two inequivalent valleys (±*K* points) at the corners of the hexagonal Brillouin zone. Owing to the extreme spatial and dielectric confinement in the single-layer limit, many-body Coulomb interactions have been found to be particularly strong compared to conventional semiconductors, as evidenced by neutral and charged exciton (trion) states with exceptionally large binding energies^1–5^. Excitonic quasiparticles dominate the absorption and emission spectra in the optical response of materials as the oscillation strength is transferred from interband transitions to resonant absorption of excitonic states. The rich excitonic physics and complex excitonic dynamics have motivated extensive studies devoted to the fundamental understanding of the novel photophysics of TMDC monolayers^6–19^. Among these, interesting features associated with trions have been explored, such as trion-induced negative photo-conductivity^17^ and long valley lifetimes up to nanoseconds^18,19^.

The enhanced Coulomb interaction and the resulting many-body effects have also triggered intense research activities when optical excitation density is increased beyond the linear-response regime (≤10^11^ excitons/cm^2^). Strong interactions between excitons have been revealed by observations of exciton-exciton annihilation and of biexcitons^20–22^. It is known that the photogeneration of excited-state carriers can enhance electronic screening and lead to a significant transient bandgap renormalization with increasing photoexcitation densities^23–30^, thus exhibiting a potential impact on the transient optical response of TMDCs-based opto-electronic devices such as photodetectors. Recent pump-probe experiments at room temperature indicate that bandgap renormalization effects need to be considered in the photoexcited carrier-density regime of >10^12^/cm^2^ ^23,26^. In terms of monolayer MoSe_2_, its photo-induced dynamics or many-body effects can be different, or even contradictory, at different excitation conditions. For example, the population transfer from excitons to trions occurs when the photoexcited carrier density is in the linear-optical response regime or when the laser excitation energy lies above the free-carrier gap^31–34^, but excitons and trions generated from free photocarriers can decay independently at the quasiresonant excitation when the photo-injected carrier density is as high as 10^12^–10^13^/cm^2^ ^35,36^. These studies have indicated that exciton and trion dynamics upon optical excitation at a relatively high carrier density are much more complex, in addition to exhibiting intrinsic radiative and nonradiative recombination. Therefore, understanding the explicit process of complex exciton and trion dynamics requires a detailed investigation of the dynamic interactions between excitons and trions, including phenomena such as exciton-energy shift, Pauli blocking, and trion formation at a low temperature as a whole.

In this work, we first accessed the relatively high-density excitation regime and mainly detected the nonlinear dynamics of optical response and valley dynamics of trions in monolayer MoSe_2_ at a temperature of 10 K, with an applied optical pumping fluence up to 40 μJ/cm^2^. By tuning the pump photon energy, excitons or trions can be selectively excited in order to investigate their coupling and decay dynamics. We observed a unique evolution process of transient differential reflectivity (Δ*R*/*R*) as a function of excitation energy and density, in which the dynamic photobleaching of trions and the redshift of the exciton resonance were observed to be attributed to the decay dynamics. Polarization-resolved differential reflectivity measurements further suggested that the initial fast population and polarization decay process observed for trions after photoexcitation was connected to trion formation, phase-space filling and the sub-picosecond intervalley scattering time of excitons resulting from strong exciton-trion correlations.

## Results

**Figure 1:**
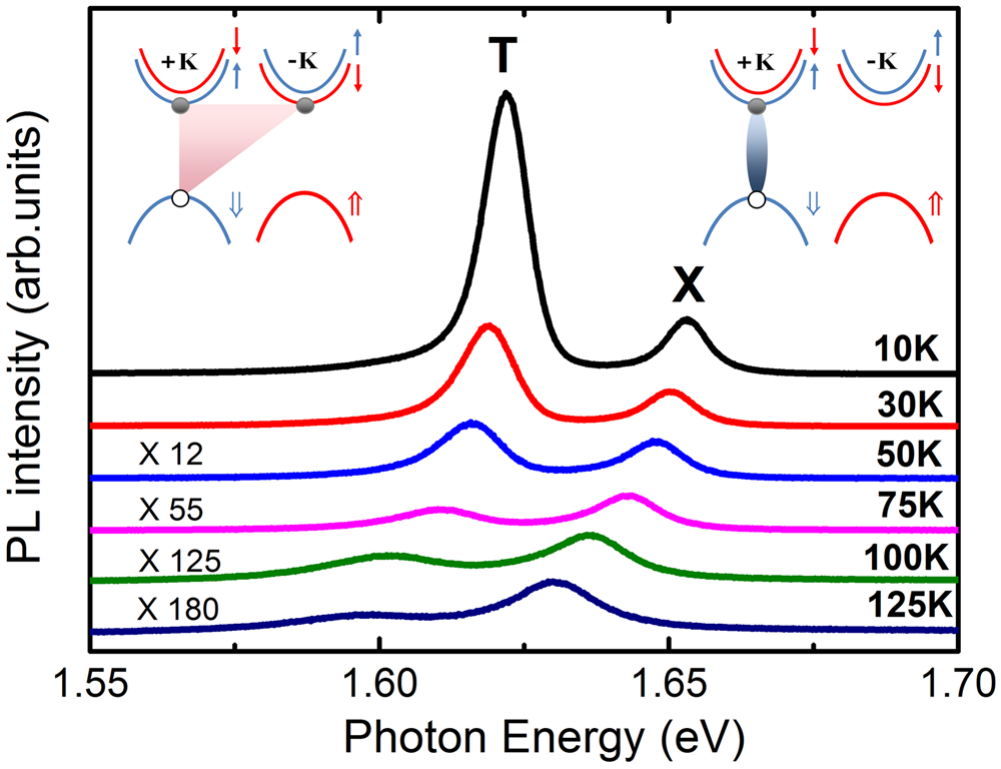
Temperature dependent photoluminescence spectra of monolayer MoSe_2_ measured in the range of 10–125 K. Exciton (X) and trion (T) peaks are labeled. Band diagram of trion (left panel) and exciton (right panel) are sketched in the inset (⇑ and ↑ represents the hole and electron spin state in valence and conduction band, respectively).

In Fig. 1, the two distinct excitonic emission peaks at 1.653 and 1.622 eV at *T* = 10 K, corresponding to the neutral A exciton and the trion, respectively, are observed for monolayer MoSe_2_^5,31^. With increasing temperature, the integrated PL intensity of trions shows a dramatic reduction by up to 100 times in the range from 10 to 100 K, in agreement with previous measurements^34,35^. The exciton is formed by the electron and hole pairs in the same momentum valley, while the negatively charged trion can be formed between a electron-hole pair in the K valley and a single spin-down electron in the -*K* valley (as displayed in the inset of Fig. 1). The stronger PL intensity of the trion peak, with respect to that of the neutral exciton, reflects the efficient trion formation in our studied sample. The well-resolved trion resonance in MoSe_2_ allows us to investigate its valley dynamics of exciton and trions independently.

**Figure 2:**
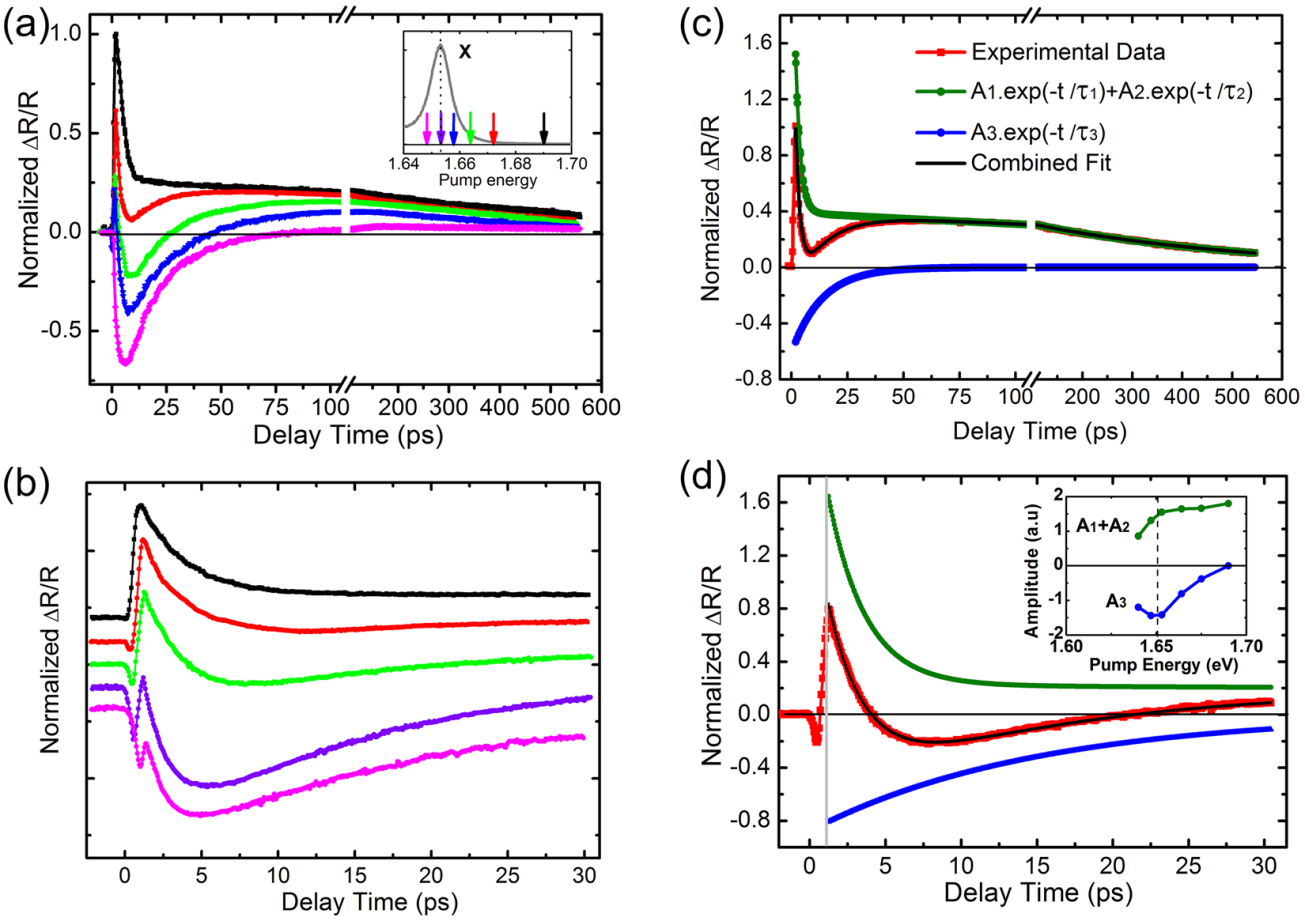
(**a**) Normalized transient reflectivity at different pump photon energies with the probe photon energy fixed at the trion resonance. The inset displays the corresponding pump energy near the exciton resonance. (**b**) The corresponding transient reflectivity within 30 ps measured with higher time-resolution. (**c**) and (**d**) The decay processes and the fit to the data obtained at the pump energy of 1.676 and 1.665 eV, respectively. The amplitudes of the positive (*A*_1_ + *A*_2_) and negative (*A*_3_) components as a function of pump photon energy are shown in the inset of (**d**).

To study the trion valley dynamics of MoSe_2_ monolayers at the applied excitation density, the transient reflectivity of trions was first investigated with the probe energy fixed at the trion resonance, but with the pump energy tuned from the high- (1.690 eV) to low-energy side (1.645 eV) of the exciton resonance, as shown in Fig. 2(a). A few selected pump photon energies (as marked by arrows) can be seen in the inset of Fig. 2(a). The dynamical evolution of the ∆*R*/*R* is complex and includes a change in sign with decreasing the pump photon energy. The detailed dynamical evolution process within the initial delay time of ~30 ps was further checked with a higher time resolution (much smaller scanning steps of the motional stage), as shown in Fig. 2(b). At a time delay of approximately 1 ps, a sign reversal feature appeared in the Δ*R*/*R* signal, which will be discussed later. The phenomena under strong optical excitation are obviously different from that of the coupling and transfer between excitons and trions observed at the low-excitation-density regime^31,32^. The complicated reflectivity dynamics can be well fitted by using a function consisting of three exponential decay components:

$${\rm{\Delta }}R/R={A}_{1}.{{\rm{e}}}^{(-t/{\tau }_{1})}+{A}_{2}.{{\rm{e}}}^{(-t/{\tau }_{2})}+{A}_{3}.{{\rm{e}}}^{(-t/{\tau }_{3})}$$

where the amplitudes of *A*_1_ and *A*_2_ are positive while that of *A*_3_ is negative. This function provides good enough fitting to all of the experimental data studied in this work, as indicated by the black lines in Fig. 2(c,d). The deduced decay time constants of the positive decay components, 2.0 ± 0.3 ps (τ_1_) and 450 ± 24 ps (τ_2_), remain nearly unchanged at the different pump energies, but the relative amplitude of short- and long-lived positive components, *A*_1_/*A*_2_, increases as the pump photon energy approaches the exciton resonance; this is seen by comparing the fitting results (olive lines) plotted in Fig. 2(c,d). In addition, the amplitude *A*_3_ of the negative decay component increases considerably, as shown in the inset of Fig. 2(d).

## Discussion

**Figure 3:**
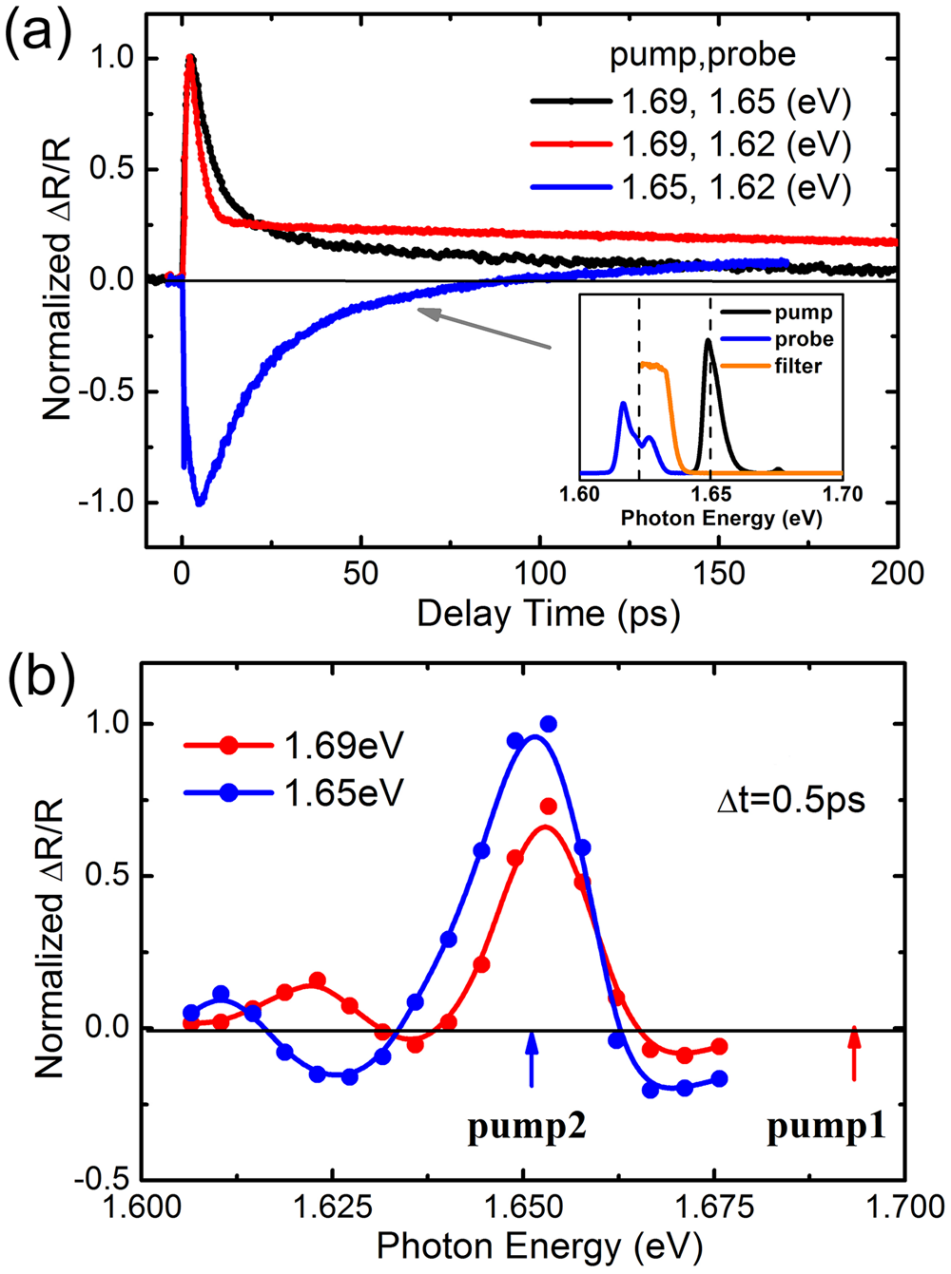
(**a**) Normalized transient reflectivity at the selected pump and probe energy. The inset shows the typical pump and probe spectrum on-resonant with excitons and trions, respectively. (**b**) Differential reflectivity spectra at Δ*t* = 0.5 ps under above- and on-resonant pumping of excitons, 1.69 and 1.65 eV, as marked by the red and blue arrows.

In order to understand the complex dynamical evolution of Δ*R*/*R* as observed in Fig. 2, the transient reflectivity was investigated by tuning the probe photon energy as well. The normalized transient Δ*R*/*R* responses were then compared for excitons and trions with the photon energy of the probe beam tuned to be resonant with the exciton and trion transition, respectively, as shown in Fig. 3(a). The pump photon energy is first set at 1.69 eV, which is higher than the A exciton transition but far below the B exciton transition. The Δ*R*/*R* response of excitons (black trace in Fig. 3(a)) is measured to decay bi-exponentially, with the deduced decay time constants being 6.2 ps (τ_1_) and 67 ps (τ_2_). The fast decay is usually attributed to radiative recombination or phonon scattering, while the slow decay is associated with the nonradiative recombination process in which excitons are easily captured by defects^8,10,37^. The Δ*R*/*R* of trions (red trace in Fig. 3(a)) also decays bi-exponentially, but with distinct time constants of 2.2 ps (τ_1_) and ~450 ps (τ_2_). Different decay time constants obtained for excitons and trions will be discussed in detail later. The probe-photon-energy-dependent transient reflectivity is then investigated at an off-resonant pumping energy of 1.69 eV, and the amplitude of Δ*R*/*R* at a time delay of Δ*t* = 0.5 ps is plotted as a function of probe photon energy as shown in Fig. 3(b) (red trace). It can be seen from the figure that the amplitude of Δ*R*/*R* is positively peaked at the resonant positions of excitons and trions, consistent with the PL. Both the exciton resonance and trion resonance are bleached without an obvious shift in energy.

The observed fast decay time τ_1_ of trions is rather close to the reported trion formation times of 2.5 ps^32^ and 1.6–2.4 ps^33^. However, the excitonic decay time τ_1_ observed is longer than the trion’s fast decay, as seen in Fig. 3(a). This discrepancy reveals that both excitons and trions are directly photogenerated following quasiresonant photon absorption^35,38^. Trions are bleached due to the enhanced free carrier capture to trions. Under near-resonant exciton excitation, excitons are generated with a sufficiently small center-of-mass momentum. Owing to the very large oscillator strength, excitons exhibit fast radiative decay in the light cone, which is comparable to the observed intrinsic radiative decay time of t ≈ 2 ps at a low temperature of 7 K^35^. In addition, a portion of excitons can experience rapid exciton-exciton and exciton-phonon scattering, and thus can also be efficiently redistributed out of the light cone^32,39^. As a consequence, the phase space of excitons is redistributed, leading to the reduced blocking of trion transitions within picoseconds^19,40^, while the longer decay time τ_2_ of trions is attributed to the population loss of trions, in which trions are more localized than excitons and have a longer nonradiative time due to their lower oscillator strength than that of excitons. From the pump-photon-energy-dependent results shown in Fig. 2(a), the increased *A*_1_/*A*_2_ value as the pump photon energy approaches the exciton resonance implies that the trion formation and relaxation process in the early stages are influenced by the enhanced phase-space filling of the optically accessible *k*-space by excitons caused by the increased exciton density.

When the pump photon energy is tuned to be resonant with the exciton transition, however, the transient reflectivity for trions is quite different from that of off-resonant pumping at 1.69 eV with the same pump fluence, as shown by the blue trace in Fig. 3(a). It is seen that the dynamical evolution of the Δ*R*/*R* signal initially shows negative amplitude within tens of picoseconds and then changes its sign at a longer delay time. Correspondingly, the probe-photon-energy-dependent transient reflectivity spectra, also recorded at a time delay of Δ*t* = 0.5 ps under on-resonant pumping of excitons, shows a different dispersive line shape in Fig. 3(b). A negative Δ*R*/*R* signal appears below the exciton resonance, rather than the positive bleaching peak of trions. It is known that an exciton can capture an extra charge to form a trion via phonon-assisted exciton-to-trion down-conversion in the low-excitation-density regime^32,33^. In addition, the coupling and transfer between excitons and trions can cause the asymmetric exciton-trion cross-peaks as previously studied^31^. However, this is not the case in the high-excitation-density regime investigated here, as evidenced by the sign change of the Δ*R*/*R* spectra in Fig. 3(b).

From Fig. 2(b), it is seen that the negative decay component becomes dominant for the evolution process of the transient reflectivity when the pump photon energy approaches the exciton resonance. The negative Δ*R*/*R* is a photoinduced absorption signal^7^, which should be ascribed to the redshift of the exciton resonance^26,28^. Band-gap renormalization, together with the phase-space filling, contributes to the bleaching at the exciton resonance and the absorption on the lower-energy side. This is evidenced by the fact that the extracted time constant (~10 ps) of the negative decay component of the transient Δ*R*/*R* signals probed at the trion resonance is comparable to that deduced by the positive decay component when probed at the exciton resonance. In fact, the transient reflectivity also exhibits a negative decay when the applied pumping fluence is doubled (~80 μJ/cm^2^) at 1.69 eV (data not shown), suggesting that the transient reflectivity change observed in Figs 2(a) or 3(a) is related to the increased exciton density under near-resonant exciton excitation. The increased amplitude *A*_3_ of the negative decay component shown in the inset of Fig. 2(d) also conforms to this feature.

**Figure 4:**
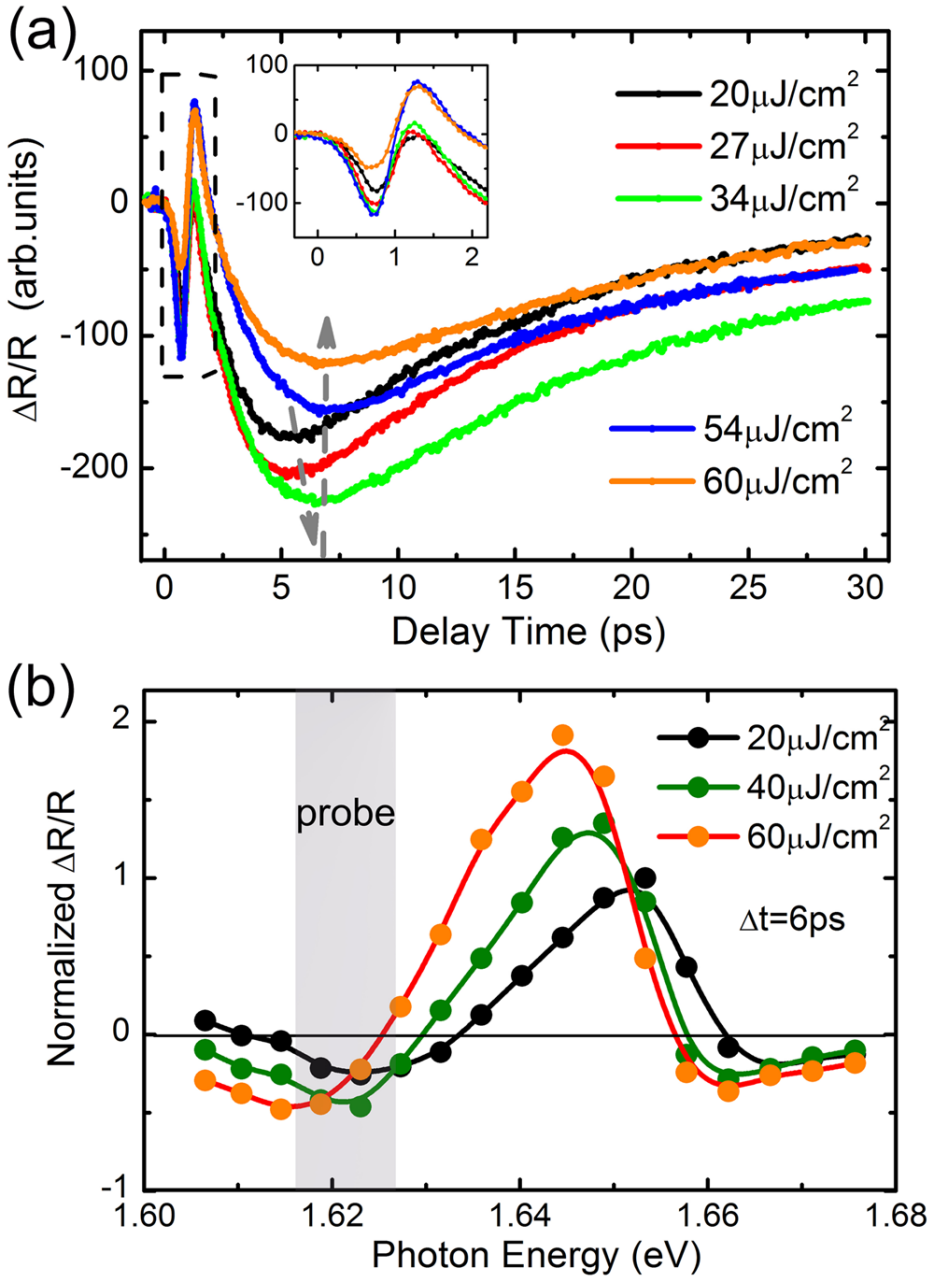
(**a**) Transient reflectivity measured at different pump fluences with the on-resonant pump (1.653 eV) at excitons and on-resonant probe (1.622 eV) at trions. The inset shows the zoom-in Δ*R*/*R* within 2 ps. (**b**) The corresponding differential reflectivity spectra at 6 ps with increasing pump fluence.

To further confirm that the negative transient component is influenced by the photo-generated exciton densities, we perform pump-fluence-dependent measurements as shown in Fig. 4(a) at the on-resonant exciton excitation. The overall amplitude of Δ*R*/*R* reaches the maximum at a delay time of Δ*t* = 6 ps as a result of the competing positive and negative transient decay components. With varying pump fluence from 20 to 60 μJ/cm^2^, the observed negative decay component first increases and then decreases. The corresponding differential reflectivity spectra are measured as a function of probe photon energy at a delay time of approximately 6 ps, as shown in Fig. 4(b). These characteristics are consistent with the enhanced band-gap renormalization effect by increasing photoinduced exciton density, which can have strong impact on the transient Δ*R*/*R* response, as shown in Fig. 4(a). With increasing pump fluence, both the energy redshift and pump-induced linewidth broadening of the exciton resonance grows, leading to the positive and negative peaks as observed in Fig. 4(b). The peak shift is approximately 20–30 meV, which is comparable to the results of a previous investigation with similar excitation densities^24^. The selected probe photon energy is fixed at an energy level of ~30 meV lower than the initial exciton resonance peak in the present low-temperature study, so that different many-body effects can be detected as a function of excitation density/photon energies. This is also different from previous investigations performed at room temperature^23,24,26^.

The transient reflectivity exhibits a sign reversal at near zero delay, as shown in Fig. 4(a). It may result from the combined contribution from the coherent exciton-trion coupling^31,32^ and the blue-detuned optical Stark effect^30^, as well as from the incoherent population decay from excitons and trions^35,39^. The coherent response appears only within the sub-picosecond pumping pulse duration, whereas the incoherent population decay remains after the pulsed excitation, which is the combined rising response of both positive and negative components due to the initial bleaching of trions and the redshifted absorption of excitons induced by the renormalization effect as discussed above. These correlated effects play an important role in the overall transient Δ*R*/*R* signals due to the finite bandwidth of the probe pulse at the trion resonance.

With a much higher pump excitation density of hundreds of μJ/cm^2^ (the carrier density is estimated to be ~10^14^/cm^2^), Chernikov *et al*.^25^ have studied the giant band-gap renormalization effect and population inversion in mono-/bi-layer WS_2_ lying in the regime of a dense electron-hole plasma beyond the Mott transition. Under the excitation density of only tens of μJ/cm^2^ conducted in this work, our results clearly show the strong optical pumping-induced modulation of the exciton and trion dynamics in monolayer MoSe_2_. The observed many-body effects resulting from strong Coulomb interactions in the atomically thin TMDC materials, are at least one order of magnitude stronger than that found in conventional quantum-well systems^40–42^.

**Figure 5:**
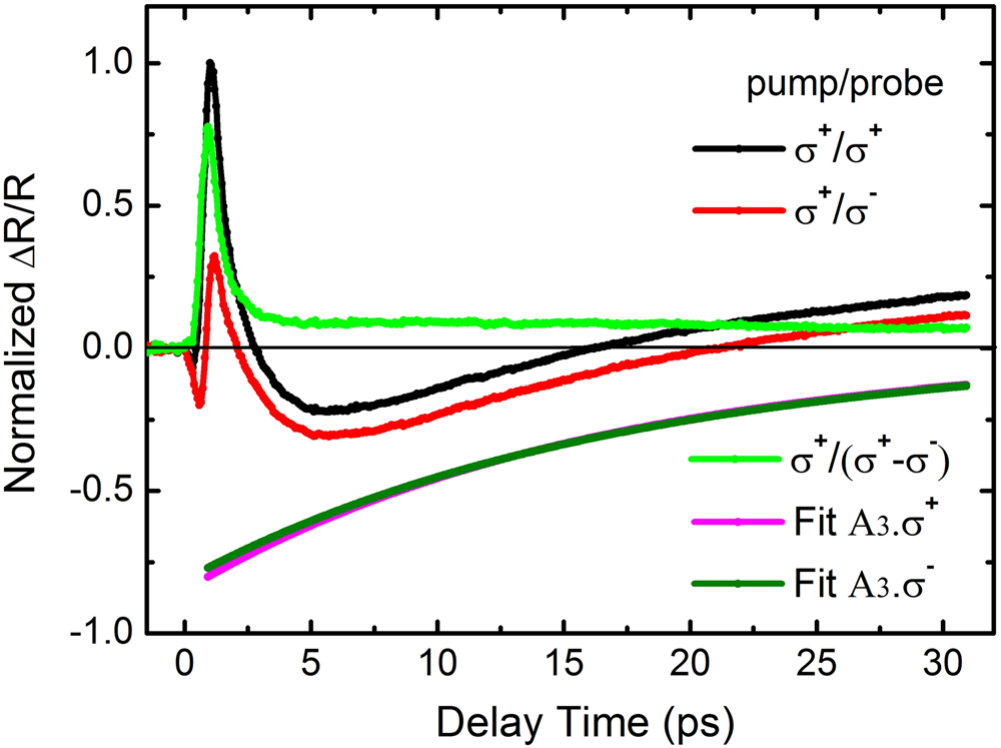
Polarization-resolved transient reflectivity of trions by σ^+^- or σ^−^-polarized probes (SCP or OCP) under σ^+^-polarized pumping. All the data are normalized relative to the peak value of SCP signal. The pump energy is set at 1.67 eV, and the probe energy is set on-resonant with trions. The green trace shows the intervalley decay dynamics by calculating the difference between the SCP and OCP signals. The negative decay components for both the SCP and OCP signals are extracted by fitting and marked with A_3_ · σ^+^ (Magenta trace) and A_3_ · σ^−^ (Olive trace), respectively.

The observed nonlinear dynamical optical responses for monolayer MoSe_2_ are expected to have an important impact on its trion valley polarization owing to the spin-valley coupling^6^. Intervalley dynamics of excitons has been extensively studied by polarization-resolved transient adsorption or reflection spectroscopy^10,28,43,44^. In this work, valley dynamics of trions in the non-equivalent +*K* and −*K* valleys is further investigated upon strong optical excitation, as shown in Fig. 5. Upon excitation of the *K* valley via right-circularly-polarized (${\sigma }^{+}$) laser pulses, the transient Δ*R*/*R* of trions recorded by the same circularly-polarized probe beam (SCP) as that of pumping reflects the photocarrier population of the *K* valley, whereas the transient Δ*R*/*R* with the left circularly-polarized probe (${\sigma }^{-}$, OCP) correlates to the rise of the population of the −*K* valley. According to the fitting results plotted in Fig. 5, the negative decay components of both the SCP and OCP signals exhibit nearly identical behavior at a probe delay time of Δ*t* > 1 ps. The valley-selective optical excitation leads to a band-gap renormalization and a redshift of the exciton resonance for both the *K* and −*K* valleys even though the −*K* valley is not optically pumped. Exciton valley polarization is then lost extremely quickly (<1 ps) due to the ultrafast intervalley scattering as a consequence of the electron-hole Coulomb exchange interaction. This ultrafast decay of the exciton valley polarization relaxation process is in good agreement with the low circular polarization degree of PL emission in monolayer MoSe_2_ reported in previous studies^45,46^.

The difference in Δ*R*/*R* response between the SCP and OCP cases comes from the positive decay components of Δ*R*/*R*. The larger amplitude of the positive decay component at a delay time of Δ*t* = 1 ps in the SCP case compared to that of the OCP case is ascribed to the enhanced phase-space filling because of the exciton and trion formation in the same *K* valley. The decay of polarization first exhibits a fast drop within a few picoseconds, followed by a long-lived decay tail, as shown by the green trace in Fig. 5. The apparent fast depolarization time constant by fitting is 0.63 ± 0.3 ps. This extremely fast initial depolarization is a consequence of the combined influence of the short exciton intervalley scattering time (<1 ps) and the exciton radiative relaxation time (~2 ps). These two kinds of valley relaxation channels, with the phase-space filling of excitons, play a major role in the initial fast depolarization when probing near the trion resonance. However, the exciton-trion correlation on the trion valley dynamics is operative only for the initial 1–2 ps, owing to the fast relaxation of excitons. Following the closing of this relaxation channel, the trion valley polarization of monolayer MoSe_2_ is observed to persist much longer, with virtually no change within the experimental observation range (~30 ps). The long valley lifetime in monolayer MoSe_2_ is in accordance with those observed in monolayer MoS_2_, WS_2_ and WSe_2_ recently, which is attributed to the spin polarization of resident carriers^19,47,48^.

## Conclusion

We have studied the nonlinear dynamics of trions in monolayer MoSe_2_ under strong optical excitation at 10 K by employing two-color transient reflection spectroscopy. Trions are directly bleached after near-resonant exciton photoexcitation due to the capture of the available free carriers to trions. In the early stage, the phase-space filling of excitons also blocks the trion transition, though this blocking only lasts for a few picoseconds due to the fast relaxation of excitons. With increasing excitation density, the redshift of the exciton resonance would induce a photo-absorption on the trion transition and greatly affect the trion valley dynamics as a consequence. These phenomena shed light on the dynamic interactions between excitons and trions and the associated many-body effects in monolayer MoSe_2_ that can stimulate a deeper understanding of the interactions of excitonic complexes in different excitation density regimes.

## Sample and Experimental Method

### Sample preparation and characterization

Monolayer MoSe_2_ samples were obtained by mechanical exfoliation of a bulk MoSe_2_ crystal (2D Semiconductors Inc.) on 285-nm SiO_2_/Si substrates. The monolayer flakes were first identified by optical contrast. A 532-nm Nd:YAG laser was then used to excite the samples, and the steady-state photoluminescence (PL) was collected by a micro-PL system. The samples were mounted on the low-vibration cold finger of a continuous flow cryostat, where the temperature could be varied between 10 and 300 K.

### Two-color pump-probe measurement

A mode-locked femtosecond Ti:sapphire laser, with a spectral linewidth of approximately 12 meV, was employed in our experiment. The output of the laser was first split into two beams. One beam served as a tunable pump and the other was used to generate a white-light continuum through the excitation of a nonlinear photonic crystal fiber (femotoWHITE-800, NKT Photonics). The tunable probe beam with a variable bandwidth can then be independently chosen by combining bandpass filters (TBP790, FF01-769/41). In order to cut off the tail of the pump spectrum that overlaps with the probe spectrum, the pump beam was first sent through a bandpass filter (FF01-747/33). The pump and probe beams were then combined collinearly and focused onto the sample. All pump-probe transient reflection measurements presented in this paper were conducted at a temperature of *T* = 10 K, with the applied pumping fluence set at ~40 μJ/cm^2^ unless otherwise noted. This corresponds to a photo-generated carrier density of 10^12^–10^13^ cm^−2^ (depending on the pump photon energy). The time delay of the probe pulse was controlled using a mechanical delay stage. We measured the relative reflection change of the probe pulse induced by the pump pulse, Δ*R*/*R* = (*R* − *R*_0_)/R_0_, where *R* and *R*_0_ were the reflectivity of the probe pulse from the sample with and without the presence of the pump pulse, respectively. Two kinds of detection schemes were used for our pump-probe differential reflectivity measurements. For the transient reflection spectra measurement in which the amplitude of Δ*R*/*R* was measured as a function of probe photon energy at a fixed time delay of the probe beam (data in Figs 3(b) and 4(b)), the reflected pump and probe beams from the sample were cross-linearly polarized in order to suppress scattering from the pump beam into a silicon photo-detector. For the time-domain dynamical two-color pump-probe measurements (data in Figs 2, 3(a), 4(a) and 5), the pump photon energy was tuned at least 10 meV higher than the probe so that the reflected pump beam from the sample can be completely filtered out by a variable bandpass filter (TBP790). All of the filters mentioned above are manufactured by Semrock, Inc. or Andover, Inc.
